# Effectiveness of Food-Based Intervention to Improve the Linear Growth of Children under Five: A Systematic Review and Meta-Analysis

**DOI:** 10.3390/nu15112430

**Published:** 2023-05-23

**Authors:** Abdullah Al Mamun, Trias Mahmudiono, Ririh Yudhastuti, Nining Tyas Triatmaja, Hsiu-Ling Chen

**Affiliations:** 1 Doctorate Degree Program in Public Health, Faculty of Public Health, Universitas Airlangga, Surabaya 60115, Indonesia; abdullah.al.mamun-2022@fkm.unair.ac.id (A.A.M.); nining.tyas.triatmaja-2022@fkm.unair.ac.id (N.T.T.); 2 Department of Nutrition, Faculty of Public Health, Universitas Airlangga, Surabaya 60115, Indonesia; hsiulinchen@mail.ncku.edu.tw; 3 Department of Environmental Health, Faculty of Public Health, Universitas Airlangga, Surabaya 60115, Indonesia; ririh-y@fkm.unair.ac.id; 4 Department of Food Safety/Hygiene and Risk Management, College of Medicine, National Cheng Kung University, Tainan 70101, Taiwan

**Keywords:** linear growth, food intervention, stunting, food based, early life nutrition, malnutrition, undernutrition, wasting, underweight

## Abstract

Food-based interventions to improve linear growth are most often applied in low- and middle-income countries. However, not all food interventions have been proven to be effective in promoting linear growth. This study aimed to assess the impact and effectiveness of food interventions for improving linear growth in children under five years old. This study was conducted by following the PRISMA guidelines and the data were extracted and presented following the PRISMA recommendations. Studies were identified through a literature search of the SCOPUS, Web of Science, PubMed, ScienceDirect, and ProQuest databases from 2000 to 2022. Only randomized control studies were included in this review based on the inclusion and exclusion criteria. Out of 1125 studies identified, a total of 15 studies were included in this systematic review and meta-analysis. The review result indicated that food-based intervention can help to improve linear growth (MD: 0.20, 95% CI: 0.04 to 0.35, *p* = 0.01) among children under five. However, there was no significant difference in changes in underweight status (MD: 0.25; CI: −0.15 to 0.64; *p* = 0.22) and wasting status (MD: 0.09; CI: −0.02 to 0.20; *p* = 0.12) between the intervention and control groups. Overall, food-based interventions were found to be helpful for improving children’s linear growth.

## 1. Introduction

Ending all types of malnutrition by the year 2030 is one of the sustainable development goals (SDGs), including, by 2025, achieving the internationally set goals for preventing stunting and wasting in children under the age of five. According to the World Health Organization’s (WHO) growth standards, stunting is defined as a height-for-age z-score (HAZ) < −2 or a length-for-age z-score (LAZ) < −2, which is the most common risk of malnutrition due to the poor growth [1]. In 2020, globally, 149.2 million (22%) children under five years old were affected by stunting and approximately 50% were from Asia [2]. In addition to increased morbidity and mortality, child stunting has both short- and long-term repercussions including poor physical and mental development with learning capacity, higher risk of infections and noncommunicable diseases in adulthood, and decreased productivity and economic capabilities [3].

According to WHO’s conceptual framework on childhood stunting, several determinants are the causes of stunting among children around the world, including poor quality foods, food and water safety, breastfeeding, household and family factors, and infectious diseases [4]. Several studies also stated that there is a significant relationship between child stunting and inadequate complementary feeding and poor-quality foods in the form of low energy content, inadequate feeding, less frequency, low dietary diversity, imbalance of food consumption from animal or plant sources, antinutrient content, etc. [3,5,6]. The most critical period of a child’s linear growth is in the first few years of life, especially 6–60 months when growth deceleration is common and necessary to meet the nutritional requirements for proper growth [4]. To meet the nutrition demand, supplementary foods are often provided to children to overcome the lack of energy content and micronutrient requirements [6].

Food intervention is often provided in different forms based on locally based family food, processed food, the priority of energy and micronutrients, and the type of food sources. In addition to improving the stunting (LAZ) condition, food intervention also showed an effect on several malnutrition conditions such as underweight (weight-for-age z-score or WAZ < −2), wasting (weight-for-length z-score or WLZ < −2), and overweight (BMI-for-age z-score or BAZ > +2) as well as other health conditions including anemia and diarrhea [7,8]. However, the effects of food intervention may differ based on several influential factors such as the baseline characteristics of the intervention group, type of nutrition, nutrient composition, intervention duration, and adherence to micronutrients [7,9].

Food supplementation is essential for a child’s linear growth as protein, micronutrients, and other food nutrients play a crucial role to achieve optimal linear growth. Food intervention also plays a significant role in many growth-preventing events including microbial exposure, infectious diseases, gut inflammation, and immunity problems [1,8]. Protein from food intervention can raise insulin-like growth factor-1 (IGF-1) concentrations; IGF-1 is a crucial growth hormone that mediates the effect of pituitary growth hormone (GH), which promotes linear growth [10]. Additionally, food intervention maintains bone integrity and gut health for linear growth among children.

Based on the background and factors causing child stunting explained above, it is important to find the effective food intervention that may help policy making and implementation of a successful nutrition program to achieve the targets of SDGs in the form of reducing the prevalence of stunting. There are several original studies on food intervention against stunting and other malnutrition problems. However, not all studies and food interventions have an equal impact in combating the stunting problem. The effectiveness of food intervention also varies based on different settings. As a result, it is important to study the effectiveness of different food interventions, types of food, intervention settings, significant findings, and any adverse effects for developing future food interventions. This study was undertaken to synthesize the findings of the effectiveness of food-based interventions to improve the stunting condition. It is expected that diversity in food intervention will help the nutritional requirement to eliminate the overall stunting graphs around the world.

## 2. Materials and Methods

### 2.1. Search Strategy

This review study followed the preferred reporting items for systematic reviews and meta-analyses (PRISMA) recommendations for review and analysis [11]. The literature search was conducted in several databases including SCOPUS, Web of Science, PubMed, ScienceDirect, and ProQuest on 16 January 2023 and thus all literature published from 2000 to 2022 was screened. The strategy used was by using synonyms of several keywords such as exposure: food intervention, food supplement, school lunch, dietary supplements, food formulated; outcome: stunting, child growth, and children linear growth; and study population: under five, child, preschool children. The complete search strategies for different database searches are detailed in Table 1. 

### 2.2. Inclusion and Exclusion Criteria

Study papers were selected based on the following inclusion criteria: (1) participant (population): studies involving children under 5 from around the world, (2) studies containing food-based intervention, either local food or processed food, (3) studies aimed to investigate the stunting condition, (4) original research papers, (5) randomized controlled trials, (6) follow-up of recent intervention, (7) minimum intervention one month, and (8) reports published in the English language.

Studies were excluded from this study based on the following criteria: (1) experimental supplement to breastfeeding mom to check development in babies, (2) intervention during pregnancy and outcome in children, (3) specific group of children affected by severe disease or health condition such as HIV, cancer, brain injury, autism, etc., (4) the study outcome focused on other interventions such as nutrition intervention, positive deviance, parenting intervention, etc., (5) formula intervention to infant during exclusive breastfeeding age, and (6) articles not available online in full version or not available in the English language.

### 2.3. Data Extraction

All search records were exported from the databases and the duplicate papers were removed. The entire process of record identification, screening, and eligibility checking was completed by AAM and rechecked by TM and NTT. The initial screening was completed by reviewing the title and abstract of all identified records to select the eligible studies that met the inclusion criteria. Thereafter, the second screening was completed to confirm the eligibility through a full-paper review of the studies selected from the first screening. Studies were excluded based on not meeting the inclusion criteria, not randomized study design, low methodological quality, having a specific group of children with a chronic disease or health condition and not having original research. 

Data were extracted from the papers that passed the eligibility criteria and included in this review study. Extracted data containing methodological and outcome variables from each study were as follows: authors, publication year, study design, participants’ age range, total sample size, intervention type, intervention components, duration of intervention, significant findings, study limitations, study outcomes, country or location, and study year. 

### 2.4. Quality of the Studies

The quality of the included studies was assessed based on the criteria adopted by The Oxford Centre for Evidence-based Medicine (https://www.cebm.ox.ac.uk (accessed on 28 February 2023)) and Fricton et al. [12]. The ten items that were considered to evaluate the quality rating of the studies selected for this review included: research questions/objectives clearly stated; the study was randomized, randomized trial, or randomized controlled trial (RCT); the method of randomization was adequate; blinding of the participants and study providers to experiment group assignment, study population/sampling frame specified/define/appropriate; similar characteristics of groups at baseline (e.g., demographics, risk factors, co-morbid conditions); the authors reported that the sample size was sufficiently large with at least 80% power (Table 2). One reviewer extracted the data and evaluated the quality of all studies and other reviewers re-checked the process for validity and completeness.

We also assessed the risk of bias (ROB) of the included reports according to the Cochrane Collaboration risk of bias tool. The assessment domain of the ROB includes the following: (1) selection bias (randomization process), (2) performance bias (deviation from intended intervention), (3) detection bias (outcome assessment), (4) attrition bias (missing outcome), (5) reporting bias (selective reporting), and (6) other bias [28]. Each study was classified as low, unclear, or high risk based on these domains.

### 2.5. Data Synthesis and Analysis

The data of the outcomes from the selected studies were synthesized for the meta-analysis. The effect size of food intervention on relevant outcomes was assessed as the (mean ± standard deviation (SD)) before and after treatment in both the intervention and control groups. 

In trials where the mean SD was divided into baseline and end-line values, certain transformations based on Cochrane’s formulae for combining groups were used to extract one mean and one SD. The following formula (Equation (1)) was used to determine the standard deviations (SDs) of the mean difference between baseline and end-line values in both the intervention and control groups:

(1) $$\mathrm{Differences}\;\mathrm{of}\;\mathrm{SD}=\sqrt{{{(SD}_{baseline}}^{2}+{{SD}_{endline}}^{2}-2R\times {SD}_{baseline}\times {SD}_{endline}}$$

Review Manager software, version 5.4.1 (Cochrane IMS, Oxford, UK), was used to conduct the meta-analysis, run statistical analysis, and create the forest plots. Statistics were deemed significant for *p*-values under 0.05. The publication bias was visualized by Review Manager software. In order to assess the publication bias of specific research, a funnel plot was used.

All the data for this research came from open-access articles and databases, so it was exempt from IRB (Institutional Review Board) at the Faculty of Public Health (namely KEPK, FKM), Universitas Airlangga.

This review study has registered as a systematic review in PROSPERO, Centre for Reviews and Dissemination, University of York (https://www.crd.york.ac.uk/prospero (16 May 2023)) with registration ID No. CRD42023421951.

## 3. Results

### 3.1. Reporting Results and Study Selection

A total of one thousand one hundred and twenty-five records were retrieved after an extensive literature search from the databases, among them 199 from Scopus, 449 from PubMed, 74 from Web of Science, 32 from ProQuest, 331 from ScienceDirect, and 40 records from a secondary manual search. After the removal of 89 records as duplicates, 1036 records were retained for primary screening by examining titles and abstracts. A further 963 records were eliminated and the remaining 73 studies met the criteria for full-text evaluation. Then, a total of 58 records were excluded according to the established inclusion and exclusion criteria for this study.

**Figure 1:**
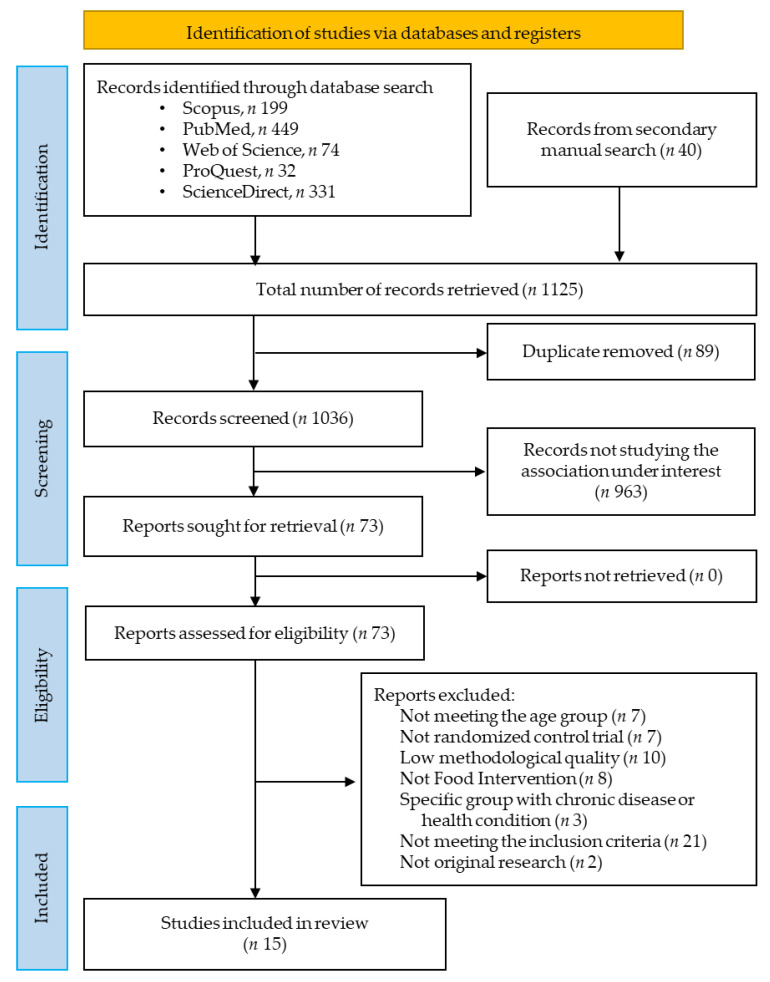
PRISMA flow diagram of the literature search and study selection for systematic review and meta-analysis.

Finally, 15 studies were selected (as shown in Figure 1) for systematic review and meta-analysis [13,14,15,16,17,18,19,20,21,22,23,24,25,26,27]. 

### 3.2. Study Characteristics

All 15 studies included in this review were randomized control studies, with a total of 15,909 participants. Table 3 lists the PICOS criteria for including and excluding studies. A summary of the general characteristics of selected studies included in this review is presented in Table 4. In addition, the types of food intervention, nutrition-related information, frequency, calories, and study findings are shown in Table 5. Different studies gave the food intervention to the participants for different durations, such as 1 month [25], 2 months [15], 5 months [24], 6 months [13,16], 7 months [19], 12 months [17,18,21,22,23,26,27], 15 months [20], and 6–18 months [14]. Most of the selected studies were from countries in Asia and Africa, whereas only two studies were from Colorado, USA. 

The quality of included studies analyzed for randomized trials and eligibility for meta-analysis. The overall quality rating (QR) of all selected studies is represented in Table 2. In 46.7% of studies, the study participants and providers were unblinded to treatment group assignment. Among selected studies, two studies did not represent the baseline data [18,26]. Two studies did not represent the output (stunting/linear growth) as LAZ score [15,17]. In addition, some studies were not included in the meta-analysis due to the fact that the data were in a different format. 

### 3.3. Risk of Bias

**Figure 2:**
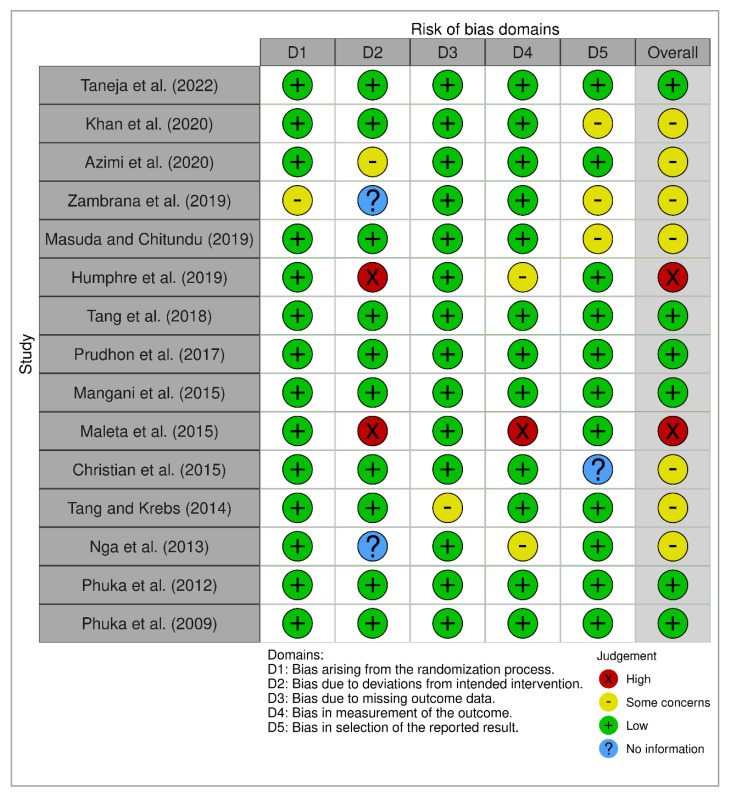
The summary of the risk of bias [13,14,15,16,17,18,19,20,21,22,23,24,25,26,27].

The result of the risk-of-bias assessment is shown in Figure 2, which includes six domains of bias. Each domain was categorized as low (+), some concern (-), high (×), and no information (?). Six studies were listed as having an overall low risk of bias [13,19,20,21,26,27], two studies were at high risk of bias [18,22], and the remaining seven studies were listed as being in the “some concern” group [14,15,16,17,23,24,25]. Almost all the studies except one [16] were rated as having a low risk of selection bias (randomization process). Two studies [18,22] showed high-risk bias in the domain of performance bias (deviation from intended intervention), whereas another two studies [16,25] did not report detailed information. Almost all of the studies listed a low risk of bias for the domain of attrition bias (missing outcome), whereas one study [24] was evaluated as having “some concern” in that domain. In the domain of detection bias (outcome assessment), one study [22] showed a high risk of bias, two studies [18,25] showed “some concern” for bias, and the remaining studies had a low risk of bias. Lastly, three studies [14,16,17] showed “some concern” and one study [23] did not report the information in the domain of reporting bias (selective reporting).

**Figure 3:**
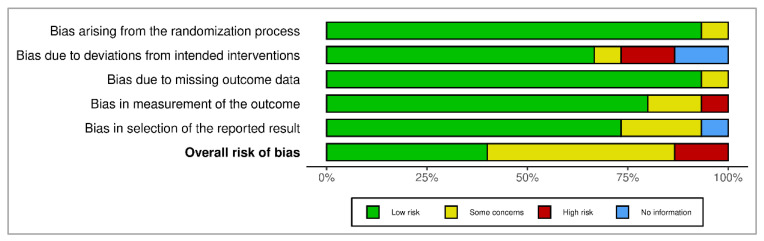
The summary of risk of bias as percentage.

The summary of ROB is presented as a percentage in Figure 3, and it shows the overall percentage of several risk of bias in different classes of domains. 

### 3.4. Meta-Analysis Results

#### 3.4.1. Primary Outcome

**Figure 4:**
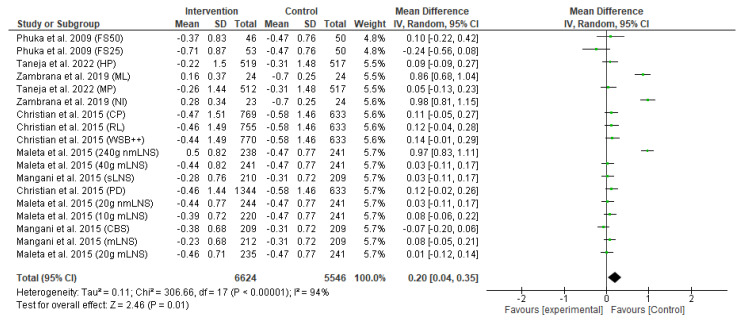
Effect of the food-based intervention on child stunting (LAZ score) [13,16,21,22,23,27]. CI: confidence interval; LNS: lipid-based nutrient supplements; nmLNS: non-milk LNS; mLNS: milk LNS; sLNS: soy LNS; MP: modest-protein group; HP: high-protein group; NI: Nicaragua (rice bran); ML: Mali (rice bran); CBS: corn–soy blend; PD: Plumpy’doz; RL: rice–lentil; CP: chickpea; FS50: fortified spread 50 g/day; FS25: fortified spread 25 g/day; WSB++: wheat–soy blend plus plus.

The primary outcome of the meta-analysis is the effect of the food-based intervention on stunting or length-for-age z-score (LAZ), which was assessed based on several RCTs. The forest plot of the LAZ score is shown in Figure 4. Pooled analysis revealed that food-based intervention led to an improvement in linear growth (MD: 0.20, 95% CI: 0.04 to 0.35, *p* = 0.01). The effect was assessed in 12,170 participants (6624 participants from the intervention group and 5546 participants from the control group). The heterogeneity between studies was high, and there was a significant between-study heterogeneity (*I*^2^ = 94%; *p* < 0.00001). The difference in heterogeneity might relate to the population differences in various studies. 

#### 3.4.2. Secondary Outcome

The secondary outcomes of this meta-analysis include the effect of the food-based intervention on underweight or weight-for-age z-score and wasting or weight-for-length z-score. 

**Figure 5:**
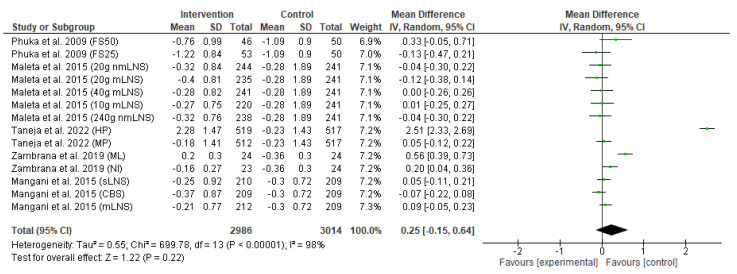
Effect of the food-based intervention on child underweight status (WAZ score) [13,16,21,22,27]. CI: confidence interval; LNS: lipid-based nutrient supplements; nmLNS: non-milk LNS; mLNS: milk-LNS; sLNS: soy LNS; MP: modest-protein group; HP: high-protein group; NI: Nicaragua (rice bran); ML: Mali (rice bran); CBS: corn–soy blend; FS50: fortified spread 50 g/day; FS25: fortified spread 25 g/day.

The forest plot of the effect of the food-based intervention on the underweight or weight-for-age z-score (WAZ) is shown in Figure 5. The analysis of effect size was assessed in 6000 participants (2986 from the intervention group and 3014 from the control group). Pooled analysis from the random-effects model indicated that there was no significant difference in change of underweight status between the intervention and control groups (MD: 0.25; CI: −0.15 to 0.64; *p* = 0.22). The heterogeneity between studies was also high (*I*^2^ = 98%), and there was a significant between-study heterogeneity (*p* < 0.00001). 

**Figure 6:**
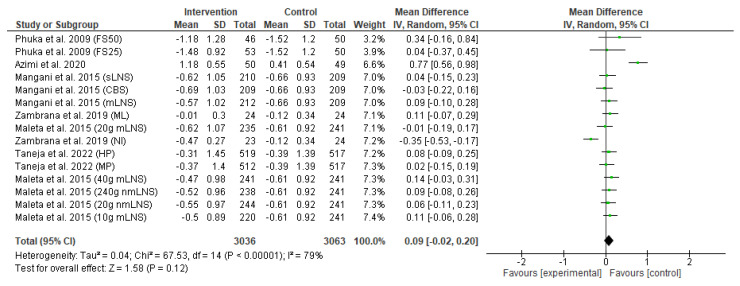
Effect of the food-based intervention on child wasting (WLZ score) [13,15,16,21,22,27]. CI: confidence interval; LNS: lipid-based nutrient supplements; nmLNS: non-milk LNS; mLNS: milk LNS; sLNS: soy LNS; MP: modest-protein group; HP: high-protein group; NI: Nicaragua (rice bran); ML: Mali (rice bran); CBS: corn–soy blend; FS50: fortified spread 50 g/day; FS25: fortified spread 25 g/day.

The effect of the food-based intervention on child wasting or weight-for-length z-score (WLZ) was assessed in 6099 participants (3036 from the intervention group and 3063 from the control group). The forest plot of effect size analysis is shown in Figure 6. Pooled analysis of random effects revealed that there was no significant improvement in child wasting between the intervention and control groups (MD: 0.09; CI: −0.02 to 0.20; *p* = 0.12). In addition, the heterogeneity between studies was also high (I^2^ = 98%; *p* < 0.00001).

### 3.5. Publication Bias (Funnel Plot)

**Figure 7:**
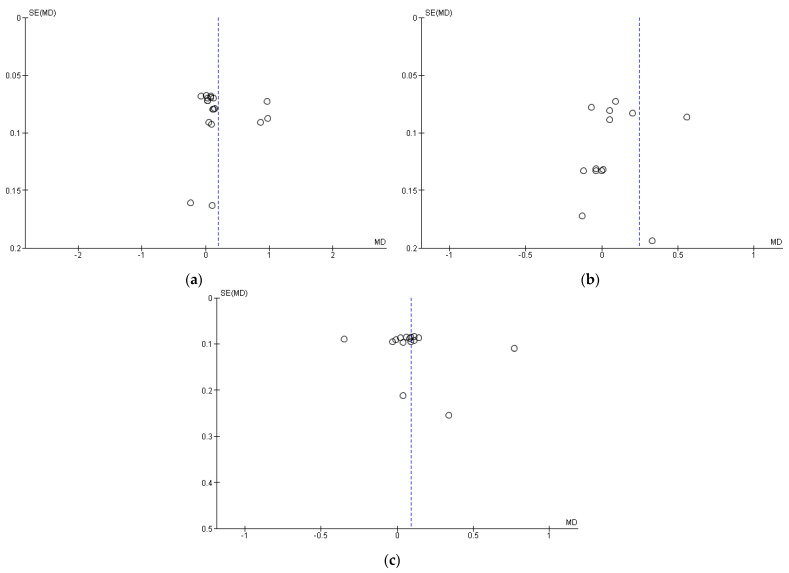
Publication bias represented as funnel plots: (**a**) Stunting; (**b**) Underweight; (**c**) Wasting.

Our meta-analysis explored publication bias by funnel plots drawn with Review Manager to assess whether any findings favored positive outcomes. The funnel plots of stunting, underweight, and wasting are asymmetrical, which indicates possible publication bias (Figure 7). 

## 4. Discussion

Stunting is still considered a major nutritional problem around the world that reflects chronic undernutrition as a failure in proper growth among children in early life [29]. Between the ages of 12 and 24 months, the frequency of stunting rises sharply (from 40% to 54%), continues to rise until the age of 36 months (58%), and then gradually stabilizes until the age of five (55%) [30]. Inadequate food and low energy and poor-quality nutrition are some important key factors that lead to stunting and in the long term; stunting may have several consequences for children, including affecting adult size, mental growth, intellectual capacity, academic performance, economic status, and reproductive capability, as well as raise the risk of metabolic disorders and cardiovascular disease [29].

In this study, we conducted a systematic review and meta-analysis to assess the effects of different food-based interventions on the improvement of linear growth. A total of fifteen research papers were included in this study based on food-based randomized trials in children under five. The minimum intervention period included in this systematic review was one month. However, a one-month intervention may not be enough to show the effectiveness of food intervention to improve linear growth [29]. It is recommended to implement food intervention for a longer duration to witness a significant reduction in the prevalence of stunting [31]. According to the meta-analysis, the overall effect of food-based interventions has a significant effect on the improve child linear growth. From the studies included in the meta-analysis, several types of foods were used in food-based interventions, including non-milk LNS, food containing rice bran, milk LNS, soy LNS, corn–soy blend, Plumpy’doz, rice–lentil, chickpea, etc., [13,16,21,22,23,27]. 

Findings from the overall systematic review revealed that both animal-source foods (ASFs) and plant-source foods are used in food-based interventions for improvement in child linear growth. Food intervention from animal sources may have a higher significant improvement in child linear growth. According to recent studies, higher protein intake from meat was associated with greater linear growth and weight gain [19,24]. Kaimila et al. also reported that food consumption with higher animal-source protein has greater effects on promoting linear growth compared with common bean or cowpea [32]. The positive impact of animal-source foods on overall health growth could be attributed to its protein quality, which is dependent on the available amino acids and its ability to be utilized by the human body. Animal-source foods are considered “high quality” because they contain sufficient amounts of essential amino acids (EAAs) that tend to be well digested or absorbed in the human body [33]. Plant protein is generally deficient in specific EAAs and less digestible.

Early childhood stunting is strongly associated with low consumption of animal-source protein, and this might be due to these sources being very expensive sources of calories, especially in low- and middle-income countries [34]. It is also recommended by researchers to readdress and promote and make animal-source proteins available to vulnerable groups and low-income countries. Dietary diversity plays a crucial role to promoting linear growth, where animal-source proteins such as meat, fish, seafood, egg, milk, and dairy products are necessary for ensuring minimum dietary diversity (MDD) [35]. The indispensable amino acid (IAA) bioavailability and possible postprandial plasma IAA concentrations of animal-source proteins are likewise significantly higher than those of plant-source proteins. However, child age, religion, family income, livelihood, and social settings play significant roles in ASF consumption [36]. Intake of ASF is important and challenging for children living in low-income settings. Considering the factors of low-income settings, commercialization of large-scale low-cost poultry production can help significantly [34]. On the other hand, small fish are a cheaper, more available, and a good source of essential nutrients compared with expensive large fish [32]. Further study also needs to be conducted to explore locally available animal source foods as the potential to increase ASF consumption. 

In addition, traditionally, some foods are used as complementary foods in intervention programs even though those foods are considered low-quality protein or poor micronutrient starchy foods such as maize, cassava, rice, and sorghum [37]. For the nearly 800 million extremely poor people who survive on less than USD 1.90/day and eat a diet heavy in starchy foods, as well as for millions of additional people who are marginally better off, more—not less—ASF will be needed for global sustainable development [38]. This is because ASF provides not only calories but, more importantly, the nutrients needed to reach physical and mental growth.

In this study, the selection of the research papers was based on stunting as the primary outcome. However, food-based intervention also has an effect on multiple health conditions including undernutrition, wasting, anemia, hemoglobin level, and diarrhea [39,40,41]. 

From Table 5, we observed that multiple studies used LNS (lipid-based nutrient supplements) as food intervention and it is important to address the concerns related to using LNS for future food intervention. The effect of LNS based on different studies yielded conflicting results. Khan et al. reported that the risk of stunting and wasting was reduced significantly among LNS recipients [14]. However, other studies concluded that the LNS supplement did not have a significant impact on linear growth [21,26], failing to promote length gain during infancy and childhood [22]. Some researchers suggest that the provision of LNS might be more appropriate in the context of food insecurity [20]. Particularly when the LNS product is used for prevention purposes, large quantities of LNS might generate serious concerns about the negative effects of excessive weight gain that may affect later life and non-communicable disease risk in the future [42]. Small-quantity LNS offers fewer calories per day (110 kcal), and the research on its effects on body composition and long-term results are still developing [43]. Additionally, sugar is added to LNS mainly to make it more palatable, and there have been some worries that this may cause babies to prefer sweet foods over breastmilk [43].

In general, stunting and other malnutrition problems are higher among low- and middle-income countries, and children with these conditions often have high infection load, poor gut health, low immunity, and gut inflammation. The types of food, especially high-quality protein, essential micronutrients, and other nutrients play significant roles in achieving optimal child linear growth because of the increased requirements due to high rates of microbial exposure, infectious diseases, and gut inflammation [13]. In addition, protein is a crucial food that has been linked to supporting children’s growth; in particular, animal-based proteins have been proven to raise insulin-like growth factor-1 (IGF-1) concentrations [10]. IGF-1 is a crucial growth hormone that mediates the effect of pituitary growth hormone (GH), which promotes linear growth. Additionally, it helps to maintain cortical bone integrity and has a GH-independent growth-stimulating impact [13]. 

Protein and amino acids are recognized as the main nutrients contributing to a child’s liner growth. Recent European feeding guidelines advise limiting early-in-life protein consumption to no more than 15% of total calories [19]. However, the exact amount of protein and energy for food supplementation for linear growth may vary depending on the baseline status of a child, such as moderately stunted or severely stunted, and their age group, such as during the age of complementary feeding or after that age. The presence of other micronutrients including zinc, vitamin D, vitamin A, calcium, iron, and iodine in food may also help in linear growth [44]. It is also suggested that several things need to be considered during designing the food intervention including the high or low biological value of protein, nutrient content of the supplemented food (especially the amino acid profile), and the protein intake from complementary food consumption. 

Evaluating the methodological quality of the selected articles indicated that some studies had some limitations that meant that they had to be excluded from the meta-analysis. Our meta-review was partly limited by the availability and types of data. Taking risk of bias into account, we tried to interpret the results very carefully. The variation in the participants in different studies may be reason for the higher heterogeneity rate in this review. The strength of this systematic review and meta-analysis study is that two reviewers independently checked and evaluated the entire data search and extraction process. There were several articles with “some concern” and “high” chance of bias based on the risk of bias analysis. In addition, we observed publication bias in studies. Moreover, the selection and use of databases for study record searches could result in sampling bias because of data mismatch concerning the hypothesis as well as lack of expected data. However, we used the PRISMA guidelines in addition to inclusion and exclusion criteria that aimed to minimize the risk of bias and increase the trustworthiness of the chosen methods. The study findings can be used in future research for planning and developing new and effective food interventions for stunting prevention. Additionally, the study findings can be considered during policy making and practice by governments, public health authorities, and other non-government organizations to design effective food interventions to eradicate overall malnutrition including stunting by promoting locally available potential food sources. 

## 5. Conclusions

According to the study findings, all the food interventions reviewed in this study had the potential to enhance a child’s linear growth. However, not all the food interventions had the same effect on the linear growth in infants and children. Challenges related to successful food intervention include consideration of nutrition quality, nutrient type, study location, local food availability, and food sources that should be taken into account for future food intervention programs. Animal protein has a great effecter on linear growth compared with plant sources. Nevertheless, more evidence is needed based on the diversity of local foods with high calorie and specific nutrient contents in the aspects of improving linear growth conditions for future practices and policies by government, non-government organizations, researchers, and nutrition-based business sectors. 

## Figures and Tables

**Table 1:** Search strategy in selected databases.

Database	Search Strategy	Filter	Records
SCOPUS	TITLE-ABS-KEY ((“Food Intervention” OR “Nutrition Intervention”) AND (“under-five” OR “child*”) AND (“Stunting” OR “linear growth” OR “child linear growth”)) AND (LIMIT-TO (SRCTYPE, “j”))	Year: 2000–2022 Research article, Language: English	199
Web of Science	(“Food Intervention” OR “Nutrition Intervention”) AND (“under-five” OR “child*”) AND (“Stunting” OR “linear growth” OR “child linear growth”)	Year: 2000–2022 Research article, Language: English	74
PubMed	#1: ((((“Dietary Supplements”[Mesh] OR “Food, Formulated”[Mesh]) OR “Food Intervention”[tw]) OR “Diet Therapy”[Mesh]) OR “Dietary Supplements/analysis”[Mesh]) OR “Dietary Supplements/statistics and numerical data”[Mesh]#2: (“Growth Disorders”[Mesh] OR Stunting[tw]) OR “Child Development”[Mesh] #3: ((“Child, Preschool”[Mesh] OR “under-five”[tw]) OR “under five”[tw]) OR child*[tw]) #4: “Counseling”[Mesh] OR “Health Knowledge, Attitudes, Practice”[Majr] ((#1 AND #2) AND #3) NOT #4	2000–2022 Randomized controlled trial, English	449
Science Direct	(“Food Intervention” OR “Nutrition Intervention”) AND (“under-five” OR “under five” OR “child”) AND (“Stunting” OR “linear growth”) NOT (“Knowledge intervention” OR “counseling intervention”)	Year: 2000–2022 Research article	32
ProQuest	(“Food Intervention” OR “Nutrition Intervention”) AND (“under-five” OR “under five” OR “child*”) AND (“Stunting” OR “linear growth” OR “child linear growth”) NOT (“counseling intervention” OR “Knowledge intervention” OR “meta-analysis” OR Review)	Year: 2000–2022 Language: English	331

* represents “truncation”, which is commonly used for article searching in several databases including WoS, PubMed, ProQuest (truncation can be used to avoid having to explicitly include all possible variants in the strategy); # represents the search number or search ID in PubMed databases. This is also common practice or strategy to use (for example, #1 OR #3 AND #3 NOT #4 to identify the previous search history).

**Table 2:** Methodological quality of the included studies.

Studies	Item 1	Item 2	Item 3	Item 4	Item 5	Item 6	Item 7	Item 8	Item 9	Item 10	Score	QR
Taneja et al. (2022) [13]	+	+	+	-	+	+	+	+	+	+	9/10	Good
Khan et al. (2020) [14]	+	+	+	+	+	+	+	+	+	-	9/10	Good
Azimi et al. (2020) [15]	+	+	+	+	+	+	+	-	+	-	8/10	Good
Zambrana et al. (2019) [16]	+	+	+	-	+	+	+	+	+	+	9/10	Good
Masuda and Chitundu (2019) [17]	+	+	+	-	+	+	+	-	+	+	8/10	Good
Humphre et al. (2019) [18]	+	+	+	+	+	+	+	+	+	-	9/10	Good
Tang et al. (2018) [19]	+	+	+	-	+	+	+	-	+	-	7/10	Good
Prudhon et al. (2017) [20]	+	+	+	-	+	+	+	+	+	-	8/10	Good
Mangani et al. (2015) [21]	+	+	+	+	+	+	+	+	+	+	10/10	Good
Maleta et al. (2015) [22]	+	+	+	+	+	+	+	+	+	+	10/10	Good
Christian et al. (2015) [23]	+	+	+	-	+	+	-	+	+	+	8/10	Good
Tang and Krebs (2014) [24]	+	+	+	+	-	-	+	+	+	-	7/10	Good
Nga et al. (2013) [25]	+	+	+	-	-	-	+	+	+	-	6/10	Fair
Phuka et al. (2012) [26]	+	+	+	+	+	+	+	+	+	-	9/10	Good
Phuka et al. (2009) [27]	+	+	+	+	+	+	+	+	+	+	10/10	Good

+: Yes, -: No; QR—Quality Rating (67% or more: Good, 34–66%: Fair); Items 1: Research questions/objectives clearly stated; 2: The study was randomized, randomized trial, or randomized controlled trial (RCT); 3: The method of randomization was adequate; 4: Blinding of the participants and study providers to experiment group assignment, 5: Study population/sampling frame specified/define/appropriate; 6: Similar characteristics of groups at baseline (e.g., demographics, risk factors, co-morbid conditions); 7: The authors reported that the sample size was sufficiently large with at least 80% power; 8: Output (stunting/linear growth) represented as LAZ-score; 9: Appropriate statistical analysis was used; 10: Data are sufficient/in a proper form for meta-analysis.

**Table 3:** The PICOS (participants, intervention, comparison, outcomes and study design) criteria for inclusion and exclusion of studies.

Parameter	Description
Participants	Studies involving children under 5 from around the world who received food intervention for growth improvement
Intervention	Food-based intervention, either local food or processed food
Comparison	Comparison with the control group without supplementation of the target food intervention
Outcomes	Improvement in child linear growth or decrease in stunting condition
Study design	Randomized controlled trials were included in this review study

**Table 4:** General characteristics of the studies included in this study.

Author (Year)	Study Design	Participant Age Group	Sample Size; Distribution	Country	Study Settings	Study Year	Trial Registry No.	Study Limitation
Taneja et al. (2022) [13]	RCT	6–12 m	1548; M: 770 (49.7%); F: 778 (50.3%)	India	Urban Delhi	NR	CTRI/2018/04/012932	Study was not completely blinded between 3 groups
Khan et al. (2020) [14]	CRT	6–23 m	870; M: 440 (50.6%); F: 430 (49.4%)	Pakistan	Local community in Thatta and Sujawal districts of Sindh	2014–2016	NCT02422953	Large difference of age of recruited children
Azimi et al. (2020) [15]	RCT	24–59 m	100; M: 49 (49%); F: 51 (51%)	Iran	Health centers	2017	IRCT2017021315536N6	Blood biomarkers could aid in a greater understanding of the RUSF’s underlying mechanisms.
Zambrana et al. (2019) [16]	RCT	6–12 m	95; M: 50 (52.6%); F: 45 (47.4%)	Nicaragua and Mali	Community health centers	2015	NCT02557373	Effects of rice bran may vary (geographical location, diet, environment, and host factors)
Masuda and Chitundu (2019) [17]	RCT	6–18 m	501; NR	Zambia	Camps	2015–2016	NCT03523182	Study was not blinded
Humphre et al. (2019) [18]	CRT	6–18 m	1777; NR	Zimbabwe	Rural districts	2012–2015	NCT01824940	NR
Tang et al. (2018) [19]	RCT	5–12 m	64; M: 30 (47%); F: 34 (53%)	Colorado, USA	Metro area	NR	NCT02142647	Intervention groups were compared with the WHO standards
Prudhon et al. (2017) [20]	RCT	6–23 m	2586; M: 1342 (51.9%); F: 1244 (48.1%)	Niger	Village area	2011–2012	NCT01828814	Selection bias by allocation by group of nearby villages
Mangani et al. (2015) [21]	RCT	6–18 m	840; M: 419 (49.9%); F: 421 (50.1%)	Malawi	Rural area	2008–2009	NCT00524446	NR
Maleta et al. (2015) [22]	RCT	5.5–6.5 m	1535; M: 58.9%; F: 41.1%	Malawi	Health center and hospital	2009–2011	NCT00945698	NR
Christian et al. (2015) [23]	CRT	6–18 m	5536; M: 49.4%; F: 50.6%	Bangladesh	Rural area	2012–2015	NR	Study was not blinded
Tang and Krebs (2014) [24]	RCT	5–6 m	45; NR	Colorado, USA	Metropolitan area	NR	NR	Sample size was relatively small
Nga et al. (2013) [25]	RCT	36–60 m	67; NR	Vietnam	Kindergarten schools	2010	NR	NR
Phuka et al. (2012) [26]	RCT	18 m	163; M: 81 (49.7%); F:82 (50.3%)	Malawi	Rural area	2005	NCT00131209	Lack of non-supplemented control group
Phuka et al. (2009) [27]	RCT	6 months old	182; M: 91 (50%); F: 91 (50%)	Malawi	Rural area	2004–2008	NCT00131209	Lack of non-supplemented control group

NR: not reported; m: months, RCT: Randomized controlled trial; CRT: Cluster randomized trial; M: male; F: Female.

**Table 5:** Type of food intervention in different studies and key findings.

Study	Intervention	Main Component	Duration	Frequency/Calorie	Significant Findings	Adverse Effect
Taneja et al. (2022) [13]	Milk–cereal mixes (2 groups: modest-protein and high-protein) Control: no food supplement	Protein, fat, and growth relevant MMN (multiple micronutrients)	6 m	1 packet/day; Modest-protein (2.5 g)∼125 kcal; High-protein (5.6 g)∼125 kcal	(1) Improvement in LAZ, WAZ, WAL, and MUAC in high-protein group (2) No significant improvement in modest-protein group	NR
Khan et al. (2020) [14]	Wawamum, a lipid-based nutrient supplement—medium quantity (LNS-MQ) Control: no food supplement	Roasted chickpeas, vegetable oil, dry skimmed milk powder, sugar, emulsifier, micronutrients, antioxidant	6–18 m	50 g/day (255 Kcal)	(1) The risk of stunting and wasting reduced significantly among Wawamum recipients (2) Significant reduction in anemia	NR
Azimi et al. (2020) [15]	Ready-to-use supplementary food (RUSF) Control: no additional food supplement, usual diet only	Soy protein isolate, whey protein, egg white, dates, vegetable oils, sugar, starch, vitamin and mineral complex	2 m	1–3 sachets per day (75 kcal/kg of body weight)	(1) Significant increase in weight and BMI (2) Greater daily height gain during the first month and improvement in WHZ (3) Lower prevalence of diarrhea and fever	No side effects throughout the study
Zambrana et al. (2019) [16]	Rice bran in food Control group: no rice bran	Rice bran without debris (rice husk, rice grain) 1 sachet > 1 g rice bran	6 m	At 6–7, 7–8, 8–10, 10–11, 11–12 months age > 1, 2, 3, 4, 5 sachet/day, respectively	(1) Daily consumption of rice bran supported changes in LAZ from 6 to 8 and 8 to12 m of age (2) WAZ was significantly improved only for Mali infants at 8 and 12 m	No adverse events were reported in the intervention group; one case of death was reported in the control group due to respiratory infection
Masuda and Chitundu (2019) [17]	Spirulina in soya-maize-based porridge Control: soya-maize-based porridge without spirulina	Spirulina in mealie meal (from maize) and soya flour porridgeControl: without spirulina	12 m	10 g spirulina + 40 g soy per day (200.6 kcal) Control: 40 g soy per day (162 kcal)	(1) HAZ and WAZ were similar in both the intervention and control groups (2) Spirulina group had lower risk of developing a cough and were more likely to be able to walk alone at 15 m (3) positive effects on upper respiratory infection morbidity prevention and motor milestone acquisition	NR
Humphre et al. (2019) [18]	Small-quantity lipid-based nutrient supplement (SQ-LNSs) Control: No food supplement	NR	12 m	1 sachet (20 g) per day	(1) Mean LAZ and hemoglobin concentration were higher than non-intervention groups (2) Intervention did not reduce the prevalence of diarrhea	One case with congenital abnormalities complained of abdominal discomfort (possibly related)
Tang et al. (2018) [19]	Meat- or dairy-based complementary foods	Dairy based (yogurt, cheese stick, whey protein) Meat based (puréed ham, puréed beef, gravy)	7 m	Total calorie intake 700 kcal/d Total protein intake 102 kcal/d (25.5 g/d)	(1) LAZ increased in the meat group and decreased in the dairy group (2) WLZ significantly increased in the dairy group	NR
Prudhon et al. (2017) [20]	Lipid-based nutrient supplements (LNS) Control: no control group	(a) Large-quantity LNS (LNS-LQ) Supplementary’Plumpy (b) Medium-quantity LNS (LNS-MQ) Plumpy’Doz	15 m	(a) LNS-LQ 92 g/day (500 kcal) (b) LNS-MQ 46 g/day (247 kcal)	(1) LNS-LQ (reference) or LNS-MQ had similar effect on incidence of severe acute malnutrition, moderate acute malnutrition, severe stunting, moderate stunting, and mortality	NR
Mangani et al. (2015) [21]	Lipid-based nutrient supplements (LNS) Control: No food supplement	Micronutrient fortified CSB or micronutrient- fortified LNS with milk protein base (milk–LNS) or micronutrient-fortified LNS with soy protein base (soy–LNS)	12 m	54 g/day of soy-LNS or milk-LNS or 71 g/day of CSB (280 kcal per day)	(1) No conclusive evidence on a relationship between the LNS supplementation and reduction of stunting	NR
Maleta et al. (2015) [22]	Milk containing LNSs or milk-free LNSs or corn–soy blend (CSB) Control: No food supplement	Soybean oil, dry skimmed-milk powder (or maltodextrine), peanut paste, micronutrients, sugar	12 m	10, 20, or 40 g/day milk containing LNSs or 20 or 40 g/day milk-free LNSs or 71 g/day CSB Calories: 55–241 kcal	(1) LNS supplementation during infancy and childhood promotes length gain or prevents stunting between 6 and 18 m of age in Malawi	NR
Christian et al. (2015) [23]	Ready-to-use supplementary foods (RUSF) and a fortified blended food Control: No food supplement	RUSF (rice–lentil, chickpea-based), Plumpy doz, wheat-soy-blend plus plus (WSB++), or Super Cereal Plus (SC+)	12 m	Plumpy’doz: 46 g or half dose; 28 g of rice–lentil and 23 g of chickpea product Calories: 125 or 250 kcal/day	(1) Deceleration in LAZ was lower in the Plumpy’doz, rice–lentil, and chickpea groups relative to control (2) WLZ decline was lower only in Plumpy’doz and chickpea groups (3) WSB++ had no significant impact (4) Stunting prevalence was 44% in control but lower by 5–6% in Plumpy’doz and chickpea groups	NR
Tang and Krebs (2014) [24]	Meat or cereal	Meat (pureed meat and gravy), iron- and zinc-fortified cereal, or iron-only-fortified cereal	5 m	Meat: total 71 g/day (equivalent to 8 g protein) Cereals: 1 serving/day (15 g)	(1) Higher protein intake from meats was associated with greater linear growth and weight gain	No adverse effects were reported during the study
Nga et al. (2013) [25]	Ready-to-use-therapeutic-foods (RUTF) in form of a compressed bar	Mung and soy beans, rice, sesame, sugar, whole milk powder, whey protein, vegetable fat, vegetable oil, and a premix	1 m	1 sachet (500–530 kcal) of RUTF/meal (total 2 meals), providing around 1000 kcal/day	(1) The nutritional status of the children improved significantly; increases in WHZ and HAZ z-scores	Nausea, vomiting, rash, and diarrhea were measured but the prevalences were too low for any statistical analysis
Phuka et al. (2012) [26]	Lipid-based nutrient supplements (LNS) or corn–soy flour (control)	Micronutrient fortified LNS spread or micronutrient fortified corn–soy flour (CSF)	12 m	CSF 70 g/day (282 Kcal) or LNS spread 25 g/day (130 Kcal) or LNS 50 g/day (264 Kcal)	(1) Daily supplementation of diet with LNS or CSF have comparable development outcomes by 18 m of age	NR
Phuka et al. (2009) [27]	Lipid-based nutrient supplements (LNS) or maize–soy flour (control)	LNS as micronutrient fortified spread (FS) or micronutrient fortified maize–soy flour	12 m	Maize–soy flour 71 g/day (282 Kcal) or FS 25 g/day (130 Kcal) or FS 50 g/day (264 Kcal)	(1) FS 50 g/day is likely to have a positive and sustained impact on severe stunting (2) Half-dose intervention may not have the same effect	NR

NR: not reported; m: months.

## Data Availability

Not applicable.
